# Identification of reference genes and their validation for gene expression analysis in phytopathogenic fungus *Macrophomina phaseolina*

**DOI:** 10.1371/journal.pone.0272603

**Published:** 2022-08-05

**Authors:** Adriana Orrego, María Cecilia Gavilán, Aníbal Arévalos, Belén Ortíz, Belén Gaete Humada, Amiliana Pineda-Fretez, María Cristina Romero-Rodríguez, María Eugenia Flores Giubi, Man Mohan Kohli, Julio C. M. Iehisa

**Affiliations:** 1 Departamento de Biotecnología, Facultad de Ciencias Químicas, Universidad Nacional de Asunción, San Lorenzo, Central, Paraguay; 2 Departamento de Química Biológica, Facultad de Ciencias Químicas, Universidad Nacional de Asunción, San Lorenzo, Central, Paraguay; 3 Cámara Paraguaya de Exportadores y Comercializadores de Cereales y Oleaginosas (CAPECO), Asunción, Paraguay; Institute for Sustainable Plant Protection, C.N.R., ITALY

## Abstract

*Macrophomina phaseolina* is a soil-borne pathogenic fungus that infects a wide range of crop species and causes severe yield losses. Although the genome of the fungus has been sequenced, the molecular basis of its virulence has not been determined. Identification of up-regulated genes during fungal infection is important to understand the mechanism involved in its virulence. To ensure reliable quantification, expression of target genes needs to be normalized on the basis of certain reference genes. However, in the case of *M*. *phaseolina*, reference genes or their expression analysis have not been reported in the literature. Therefore, the objective of this study was to evaluate 12 candidate reference genes for the expression analysis of *M*. *phaseolina* genes by applying three different fungal growth conditions: a) during root and stem infection of soybean, b) in culture media with and without soybean leaf infusion and c) by inoculating a cut-stem. Based on BestKeeper, geNorm and NormFinder algorithms, *CYP1* was identified as the best recommended reference gene followed by *EF1β* for expression analysis of fungal gene during soybean root infection. Besides Mp08158, *CYP1* gene was found suitable when *M*. *phaseolina* was grown in potato-dextrose broth with leaf infusion. In the case of cut-stem inoculation, Mp08158 and Mp11185 genes were found to be most stable. To validate the selected reference genes, expression analysis of two cutinase genes was performed. In general, the expression patterns were similar when the target genes were normalized against most or least stable gene. However, in some cases different expression pattern can be obtained when least stable gene is used for normalization. We believe that the reference genes identified and validated in this study will be useful for gene expression analysis during host infection with *M*. *phaseolina*.

## Introduction

*Macrophomina phaseolina* (Tassi) Goid. is a soil-borne pathogenic fungus that infects a wide range of plant species [1] and causes severe yield losses [2]. Disease control measures such as irrigation, crop rotation, chemical and biological control, etc. have been utilized with limited effectiveness [1]. Therefore, genetic resistance to the disease involving interaction between the host plant and *M*. *phaseolina* is considered crucial. Although its genome has been sequenced [3–5], information about the molecular pathogenic mechanisms of *M*. *phaseolina* remains scarce.

It has been reported that during the fungal infection process, several transcriptome changes take place, especially in the genes coding secreted proteins involved in pathogenesis [6–8]. In order to monitor changes in the gene expression during pathogenesis in a sensitive and specific manner, reverse transcription quantitative polymerase chain reaction (RT-qPCR) can be used. At the same time to ensure reliable gene quantification, the expression level of target genes needs to be normalized to the expression of a reference gene. A reference gene must be an endogenous gene with stable expression under specific experimental condition [9].

Traditionally, housekeeping genes such as β-actin, glyceraldehyde-3-phosphate dehydrogenase (*GAPDH*), α- and β-tubulin (*α-TUB* and *β-TUB*), hypoxanthine phosphoribosyl transferase (*HRPT*), and 18S and 28S ribosomal RNA have been used in different organisms due to their higher expression in all kind of cells and tissues and often their expression were considered stable [10–12]. Given that their expression is not always stable among different tissues and experimental conditions [10–12], reference genes for normalization of RT-qPCR data must be experimentally validated for particular tissues or cell types and specific experimental designs [9]. For this purpose, the stability of candidate reference genes expression is usually evaluated using algorithm such as geNorm [13], BestKeeper [14] and NormFinder [15].

Microarray and high-throughput RNA sequencing (RNA-seq) based transcriptomic data have also been used to identify potential reference genes other than the traditional housekeeping genes [16–18]. Although the traditionally used housekeeping genes are usually found among the stable genes, more stable reference genes have also been discovered by transcriptomic analysis [18, 19].

In fungi, several genes such as *β-TUB*, *GAPDH*, translation elongation factors *EF1-α* and *EF1-β*, have been tested to select a reference gene [20–22]. However, reference genes for *M*. *phaseolina* gene expression analysis have not been reported. In this study, traditional housekeeping genes and potential reference genes selected from previously published transcriptomic data [5, 23] were evaluated for expression analysis of *M*. *phaseolina* during root and stem infection of soybean as well as in the culture media with and without soybean leaf infusion. The efficiency of the selected reference genes was also verified by expression analysis of two cutinase coding genes.

## Materials and methods

### Fungal strains

*M*. *phaseolina* strains C9 and Nar were isolated from field-cultivated soybean plants (*Glycine max* L. [Merr.]) grown in Campo 9 (Department of Caaguazú) and in Naranjal (Department of Alto Paraná), Paraguay, respectively. Molecular identification of both strains was performed by Sanger sequencing part of Internal Transcribed Spacer 1 (ITS 1) using ITS-1 (5’-TCCGTAGGTGAACCTGCGG-3’) and ITS-4 (5’-TCCTCCGCTTATTGATATGC-3’) primers [24] at Macrogen Korea (Seoul, Republic of Korea). Sequences of C9 and Nar were deposited in the GenBank under the accession number OM802448 and OM802449, respectively.

### Identification of candidate reference genes from publicly available transcriptomic data

Publicly available transcriptomic data of *M*. *phaseolina* were downloaded from National Center for Biotechnology Information (NCBI). Reads of the BioProjects PRJNA428521 [5], PRJNA524935 [23] and PRJNA326815 (unpublished data) (S1 Table) were mapped on the genome of *M*. *phaseolina* strain MS6 [3], obtained from MycoCosm (https://mycocosm.jgi.doe.gov/) using the software STAR v2.5.3 [25]. The number of mapped reads per gene was obtained using *featureCounts* of the package Subreads v2.0.0 [26]. Expression data were normalized by Trimmed Mean of *M* values method, TMM [27], using the package edgeR [28] of the software R.

### *In vitro* root inoculation method

*In vitro* root inoculations were conducted by modifying a previously developed method [29]. Briefly, seeds of soybean cultivar A5009 were surface disinfested with 70% ethanol and 2.5% sodium hypochlorite (NaClO). After rinsing with sterile distilled water, seeds were placed on wet paper in a Petri dish for germination during 72 h at 24–26°C under darkness. Germinated seeds with root length of 0.5 to 1.5 cm were transferred to a plastic Petri dish with Hoagland’s solution (Sigma-Aldrich, Germany), solidified with 0.8% agar. The radicle was inserted into the medium through a small hole in the Petri dish under sterile conditions, allowing the shoot growth outside the Petri dish (S1 Fig). Seedling were grown under long day condition (16 h light and 8 h darkness) at 24–26°C.

After 48 h, the middle portion of primary root was inoculated with 50 uL of an aqueous suspension containing 22.5 ± 5.0 microsclerotia of *M*. *phaseolina* strains (Nar or C9). The inoculum was prepared as follows: a plug of Potato Dextrose Agar (PDA) colonized with isolate of *M*. *phaseolina* was placed on PDA containing sterile cellophane membrane on its surface and incubated at 30°C for 5 to 7 days until complete fungal growth. Then, the fungus was removed from cellophane membrane, placed in 25 mL of sterile water and vigorously vortexed several times. This suspension was kept at room temperature for 3 minutes and the supernatant was transferred to a new tube to eliminate large fungal fragments.

The infection process was carried out under long day condition (16 h light/8 h darkness) at 28°C. A 3 cm long portion of the infected primary root (1.5 cm above and 1.5 cm below the inoculation point) was collected for RNA extraction at 2 and 4 days post-inoculation (dpi). A pool of five infected roots constituted each biological replicate. *M*. *phaseolina* isolates grown on Hoagland-agar containing cellophane membrane on its surface was used as non-inoculated fungal control. Each treatment (group) consisted of at least three biological replicates besides uninfected soybean roots, which were also collected as negative control. In total 21 RNA extractions (infected group: 2 strains x 2 times x 3 biological replicates = 12 extractions; control group: 2 strains x 1 time x 3 biological replicates = 6 extractions; negative control (non-infected roots): 1 time x 3 biological replicates = 3 extractions) were performed. The total number of plates prepared for sampling was 105 (21 x 5).

### Fungal growth on potato dextrose broth (PDB) with soybean leaf infusion

A fungal plug of each isolate on PDA (8 mm diameter) was grown in 50 mL of PDB medium prepared with an infusion of soybean leaves from cultivar A5009. The leaf infusion was prepared by utilizing 20 g of young leaves (V3-V4 stage) per liter of distilled water, which was autoclaved (20 min at 121°C) and filtered. This infusion was used instead of distilled water to prepare PDB medium (Condalab, Spain). The fungal isolate grown in PDB without infusion was used as control. After 6 days of fungal growth at 30°C in dark condition, vacuum filtrated fungus was used for RNA extraction. Four biological replicates were obtained from each growth condition (with and without leaf infusion). Therefore, resulting in a total of 16 RNA extractions (2 strains x 2 growth conditions x 4 biological replicates). The same number (16) of growth media was also prepared.

### Cut-stem inoculation method of soybean

Four-week-old V2 stage soybean plants of cultivar Sojapar R42 (INBIO-IPTA, Paraguay) were used for cut-stem inoculation [30]. A plug of PDA colonized with *M*. *phaseolina* (isolate Nar) was obtained by inserting the open end of a 200 μL pipette tip into the margin of fungal culture. This plug-containing pipette tip was placed on stems, cut 25 mm above the unifoliate node (S2A Fig). Inoculated plants were maintained at long day condition at 28°C and 80% humidity. Three days after inoculation, the pipette tips were removed from each plant (S2B Fig). The infected stem of each plant was cut at 3 and 6 dpi and collected for RNA extraction. At least three biological replicates were obtained for analysis, resulting in nine RNA extractions (1 strain x 2 times x 3 biological replicates = 6 extractions, and negative control (non-infected plants): 1 strain x 1 time x 3 biological replicates = 3 extractions). A total number of nine plants were used.

### RNA extraction and cDNA synthesis

Collected samples were ground with a mortar and pestle in liquid nitrogen. Total RNA was extracted using TRIzol^®^ reagent (Thermo Fisher Scientific, USA) according to the manufacturer’s instruction, and then DNase I (New England Biolabs, USA) treatment was performed. For cDNA synthesis, 1 μg of total RNA, oligo(dT)_20_ and M-MuLV Reverse Transcriptase (New England Biolabs) were used. To assess the absence of genomic DNA, RT minus mix was prepared following the same procedures for cDNA synthesis but without M-MuLV Reverse Transcriptase enzyme. All synthetized cDNAs and RT minus mix were diluted 1:6 prior to RT-qPCR.

### Quantitative real-time PCR and data analysis

RT-qPCR was performed using 5 μL of 2x SsoAdvanced Universal SYBR Green Supermix (Bio-Rad, USA), 500 nM of gene specific primers (S2 Table) and 1 μL of diluted cDNA (or diluted RT minus mix) in a final reaction volume of 10 μL. Amplification and quantification was performed in StepOnePlus^™^ Real-Time PCR System (Thermo Fisher Scientific) with following thermal cycling protocol: 1 cycle of 95°C for 30 s and 40 cycles of 95°C for 15 s and 60°C for 15 s. Specificity of amplification was verified by melt curve analysis (from 65 to 95°C).

PCR efficiency of all genes was determined using a pool of cDNA obtained mixing equal amount of cDNA of each fungal sample of all culture conditions. Serial 10-fold dilutions were used as template for RT-qPCR to determine the slope of the regression between Cq (quantification cycle) or Ct (threshold cycle) values and the log values of each dilution. Each serial dilution consisted of three technical replicates. PCR efficiency of each primer set is shown in S2 Table. The expression stability of the candidate reference genes were evaluated using BestKeeper [14], geNorm [13] and NormFinder [15] algorithms.

Expression stability of 12 candidate reference genes was analyzed using BestKeeper, geNorm and NormFinder algorithms. BestKeeper measures gene expression stability as: 1) the standard deviation (SD) of Cq values of a given gene, and 2) Pearson correlation coefficient (*r*) between each gene and the BestKeeper index determined by the pair-wise correlation analyses between all possible combinations of analyzed genes [14]. Genes with higher values of correlation coefficient and lower values of SD are considered as stable in their expression.

By pair-wise comparisons, geNorm algorithm determines the stability value *M*, where lower value indicates higher stability. Unlike geNorm, NormFinder evaluates the gene expression stability by analyzing the overall expression variation of the candidate genes and also variation between the sample sub-groups (intra- and inter-group variations) [15]. Lower stability value determined by NormFinder is indicative of higher expression stability.

For validation of selected reference genes, RT-qPCR of two cutinase genes were performed using following primer sets: 5’-GTCAAGATTCCGGCTCGACT-3’ and 5’-ACCCTCCAAGAGCAGATACG-3’ for MPH_04379 and 5’-CCCTGCTGATATGGCTGGTA-3’ and 5’-TATCTGGGCATTTGGACACA-3’ for MPH_09279. The samples used for analysis of candidate reference genes were also used for validation. The relative expression levels of target genes were calculated using the 2^-ΔCt^ method [31, 32].

### Statistical analysis

For analysis of transcriptomic data, the stability of expression of the genes was evaluated calculating the variance of TMM-normalized read counts among the 20 samples listed in S1 Table. Mean expression level was also calculated for each gene. Those genes with mean expression level greater than the upper quartile were considered as highly expressed genes.

Differences in the expression levels of cutinase coding genes were statistically tested by one-way ANOVA (analysis of variance) followed by Tukey’s HSD (honestly significant difference) test using the package ‘*agricolae*’ version 1.3.3 [33] of the software R for expression data from *in vitro* root infection and *M*. *phaseolina* grown in PBD. Expression data from cut-stem inoculation, two-tailed Student’s *t*-test was applied using Microsoft Excel 2016. All plots were generated using the R package ‘*ggplot2*’ version 3.3.3 [34].

## Results

### Selection of candidate reference genes

Six candidate reference genes were selected based on homology to those evaluated for *Fusarium graminearum* [20] (Table 1). Publicly available RNA-seq data (S1 Table) were also re-analyzed to search for additional candidate genes. The selection of additional six genes (Table 2) was based on the following criteria: 1) higher expression level (greater than third quartile), 2) least variable expression level (lower variance) across conditions of fungal growth (S1 Table), and 3) involved in essential cellular process.

**Table 1:** List of candidate reference genes homologous to those evaluated in *F*. *graminearum*.

Gene	Description	*F*. *graminearum* locus	*M*. *phaseolina* locus	%identity^a^
*EF1β*	Eukaryotic translation elongation factor 1 β	FGSG_01008	MPH_03132	66.5
*UBC*	Ubiquitin conjugating enzyme	FGSG_10805	MPH_13012	97.3
*EF1α*	Eukaryotic translation elongation factor 1 α	FGSG_08811	MPH_05497	87.5
*β-TUB*	β-tubulin	FGSG_09530	MPH_11587	92.6
*CYP1*	Cyclophilin 1	FGSG_07439	MPH_07956	71.2
*CYP2*	Cyclophilin 2	FGSG_00777	MPH_09910	72.9

^a^Homology search based on BLASTp algorithm at the MycoCosm web portal.

**Table 2:** List of genes selected based on publicly available RNA-seq data.

Gene^a^	Description	Mean expression level	Variance
Mp05201	Vps9 domain-containing protein	6.709	0.028
Mp09417	26S proteasome non-ATPase-like protein regulatory subunit 2	8.003	0.031
Mp11185	ANTH domain-containing protein	6.747	0.041
Mp09987	HECT domain-containing protein	7.131	0.047
Mp06465	Eukaryotic peptide chain release factor GTP-binding subunit	7.445	0.052
Mp08158	14-3-3 protein	9.441	0.077

^a^For simplicity, the prefix MPH_ of the *M*. *phaseolina* gene locus tag is replaced by Mp for candidate reference genes.

### Expression profiles of 12 candidate reference genes

**Fig 1:**
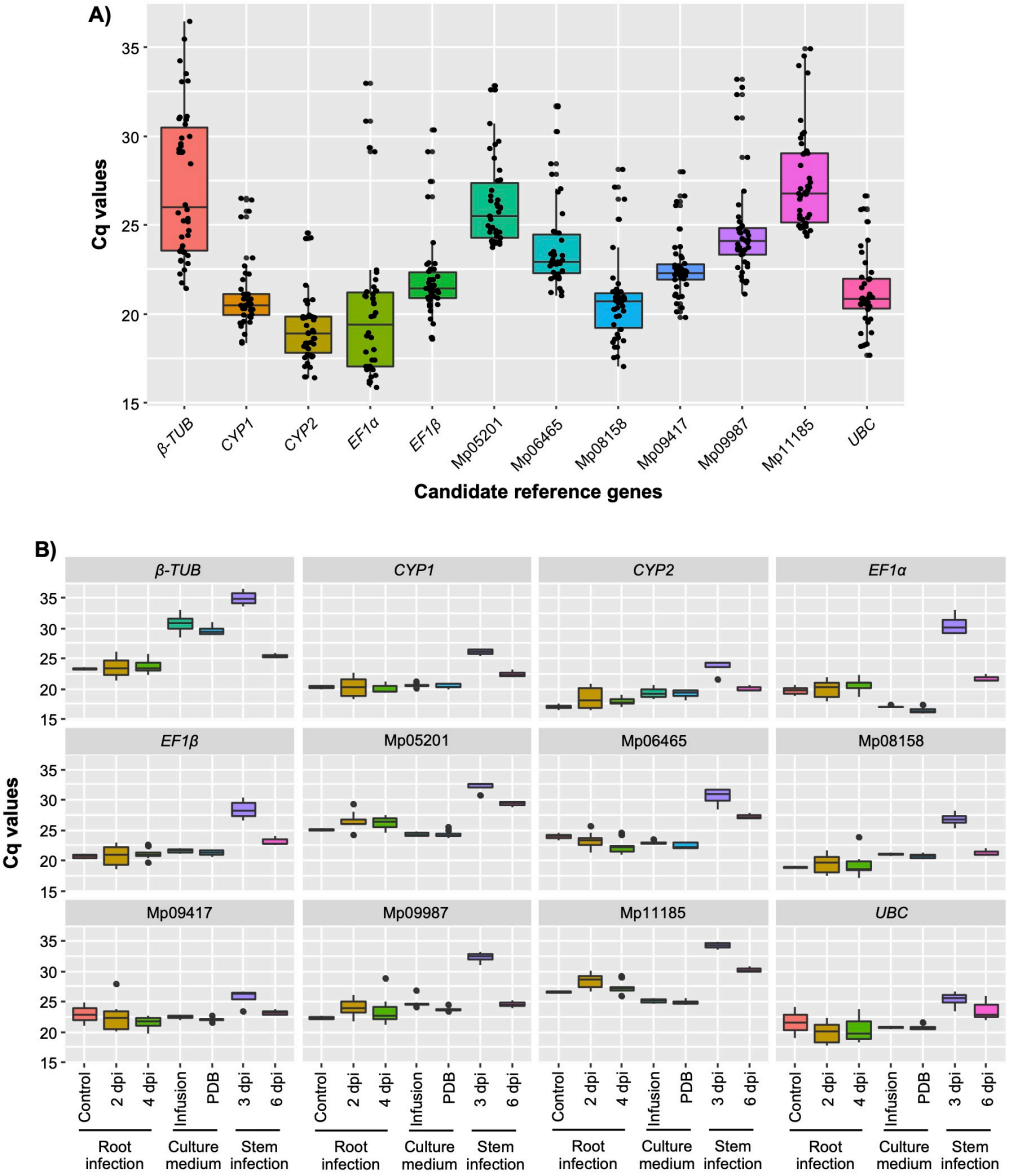
The Cq values of 12 candidate reference genes of *M*. *phaseolina* under three procedures. Box plot of the Cq values of (A) all samples, and (B) each treatment group. Box indicates the 25^th^ and 75^th^ percentiles, the central horizontal line across the box represents the median of the samples. The bars indicate the maximum and minimum values.

The median Cq values of the 12 candidate genes ranged from 18.91 for *CYP2* to 26.79 for MPH_11185 (Fig 1A). The samples used in this study can be grouped into three main categories: *in vitro* root infection, PDB with leaf infusion and cut-stem infection. In general, Cq values of *in vitro* infection and PDB with leaf infusion were lower than cut-stem infection group (Fig 1B). In this latter, samples obtained at 3 dpi presented the highest Cq values.

### Evaluation of gene expression stability

For *in vitro* root infection method, *CYP1* gene presented highest stability followed by *β-TUB* (Table 3), while *UBC* was the least stable gene. For growth in PDB with leaf infusion, Mp09417 was the most stable gene followed by *CYP1* and Mp08158, while *β-TUB* was the least stable gene. In the case of cut-stem inoculation method, Mp05201 was identified to be the most stable gene followed by Mp09417, while *β-TUB* was the least stable gene.

**Table 3:** Expression stability of candidate reference genes as determined by BestKeeper.

	*In vitro* root infection			PDB with leaf infusion			Cut-stem inoculation		
Rank	Gene	SD	*r*	Gene	SD	*r*	Gene	SD	*r*
1	*CYP1*	0.92	0.95	Mp09417	0.26	0.82	Mp05201	1.47	0.95
2	*β-TUB*	0.96	0.94	CYP1	0.26	0.71	Mp09417	1.52	0.93
3	Mp05201	0.96	0.73	Mp08158	0.28	0.82	*UBC*	1.54	0.68
4	*EF1β*	0.99	0.94	UBC	0.21	0.55	*CYP1*	1.76	0.94
5	*CYP2*	1.00	0.94	Mp11185	0.30	0.67	Mp06465	1.78	0.97
6	*EF1α*	1.06	0.83	*EF1β*	0.32	0.71	*CYP2*	1.91	0.98
7	Mp11185	1.07	0.87	Mp06465	0.36	0.77	Mp11185	1.93	0.98
8	Mp06465	1.10	0.57	Mp05201	0.37	0.42	*EF1β*	2.58	0.98
9	Mp08158	1.23	0.78	*EF1α*	0.39	0.85	Mp08158	2.68	0.99
10	Mp09417	1.34	0.82	Mp09987	0.60	0.69	Mp09987	3.81	0.98
11	Mp09987	1.45	0.81	*CYP2*	0.64	0.42	*EF1α*	4.34	0.99
12	*UBC*	1.64	0.70	*β-TUB*	1.11	0.61	*β-TUB*	4.63	0.99

**Fig 2:**
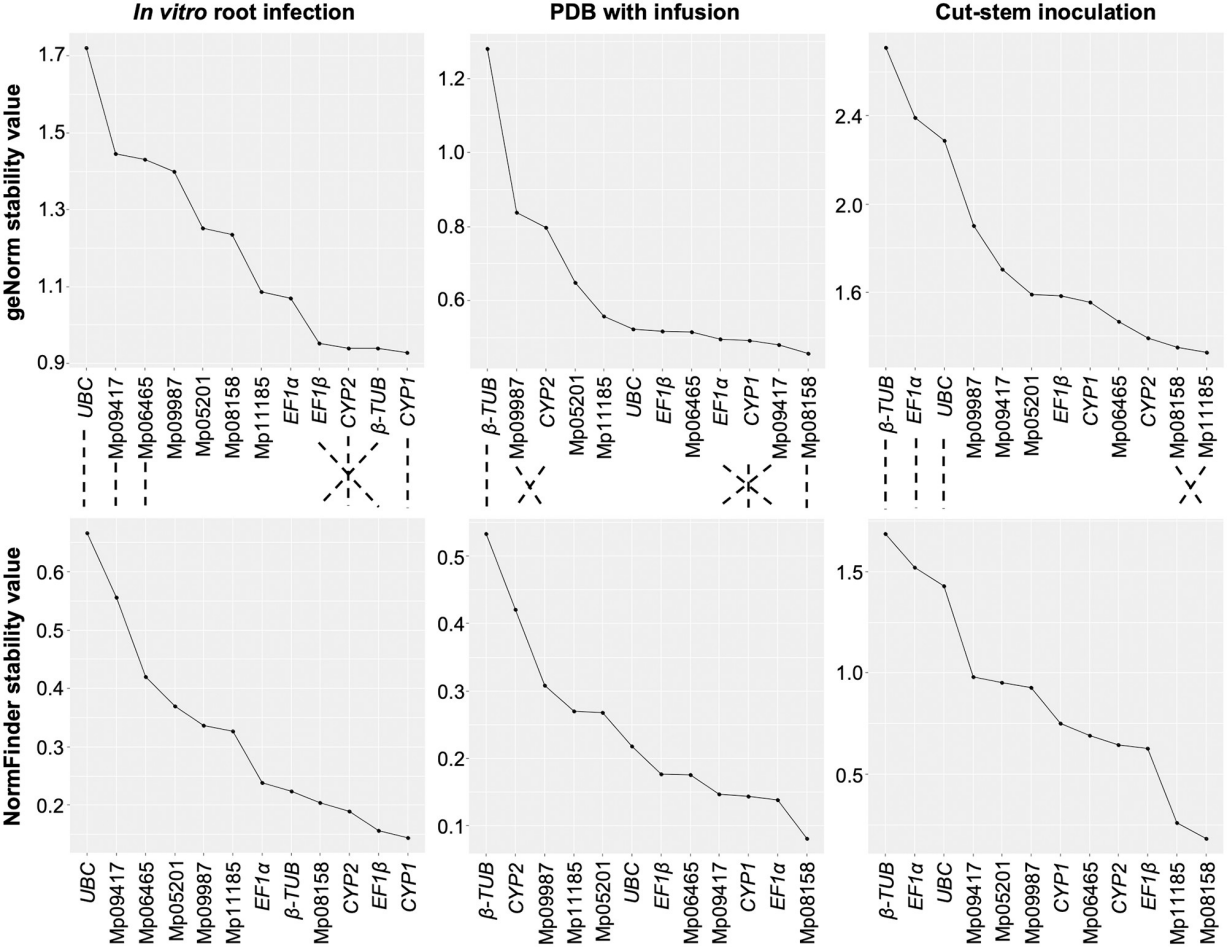
Expression stability of 12 candidate reference genes in *M*. *phaseolina* evaluated by geNorm and NormFinder algorithms. Stability of *M*. *phaseolina* genes in infected soybean roots (*in vitro* root infection method) (left), growth in PDB with leaf infusion (middle) and infected soybean stems (cut-stem inoculation method) (right) were determined using geNorm (top) and NormFinder (bottom) algorithms. Only the most and least stable genes between the two algorithms are connected by lines.

For *in vitro* root infection method, *CYP1* gene presented the highest stability when analyzed with geNorm (Fig 2). While the stability of *β-TUB*, *CYP2* and *EF1β* was similar to *CYP1*, *UBC* was observed to be the least stable gene. For growth in PDB with leaf infusion, Mp08158 was the most stable gene, followed by Mp09417, *CYP1* and *EF1α* gene (Fig 2). *β-TUB* was found to be with most variable expression. For cut-stem inoculation method, Mp11185 gene was the most stable, followed by Mp08158 gene, while *β-TUB* was the least stable gene (Fig 2).

For *in vitro* root infection method, *CYP1* showed the highest stability with NormFinder followed by *EF1β*, and *UBC* being the least stable gene (Fig 2). For growth in PDB with leaf infusion, Mp08158 presented the highest expression stability, and *β-TUB* the highest variation (Fig 2). For cut-stem inoculation method, Mp08158 was the most stable gene followed by Mp11185, while the least stable gene was *β-TUB* (Fig 2).

### Expression analysis of cutinase genes

To validate the reference genes identified for each of the three sample groups, expression of two cutinase genes (MPH_04379 and MPH_09279), considering their role in the infection process, were analyzed. Expression level of the cutinase genes were normalized using the two most stable and the least stable genes identified previously.

**Fig 3:**
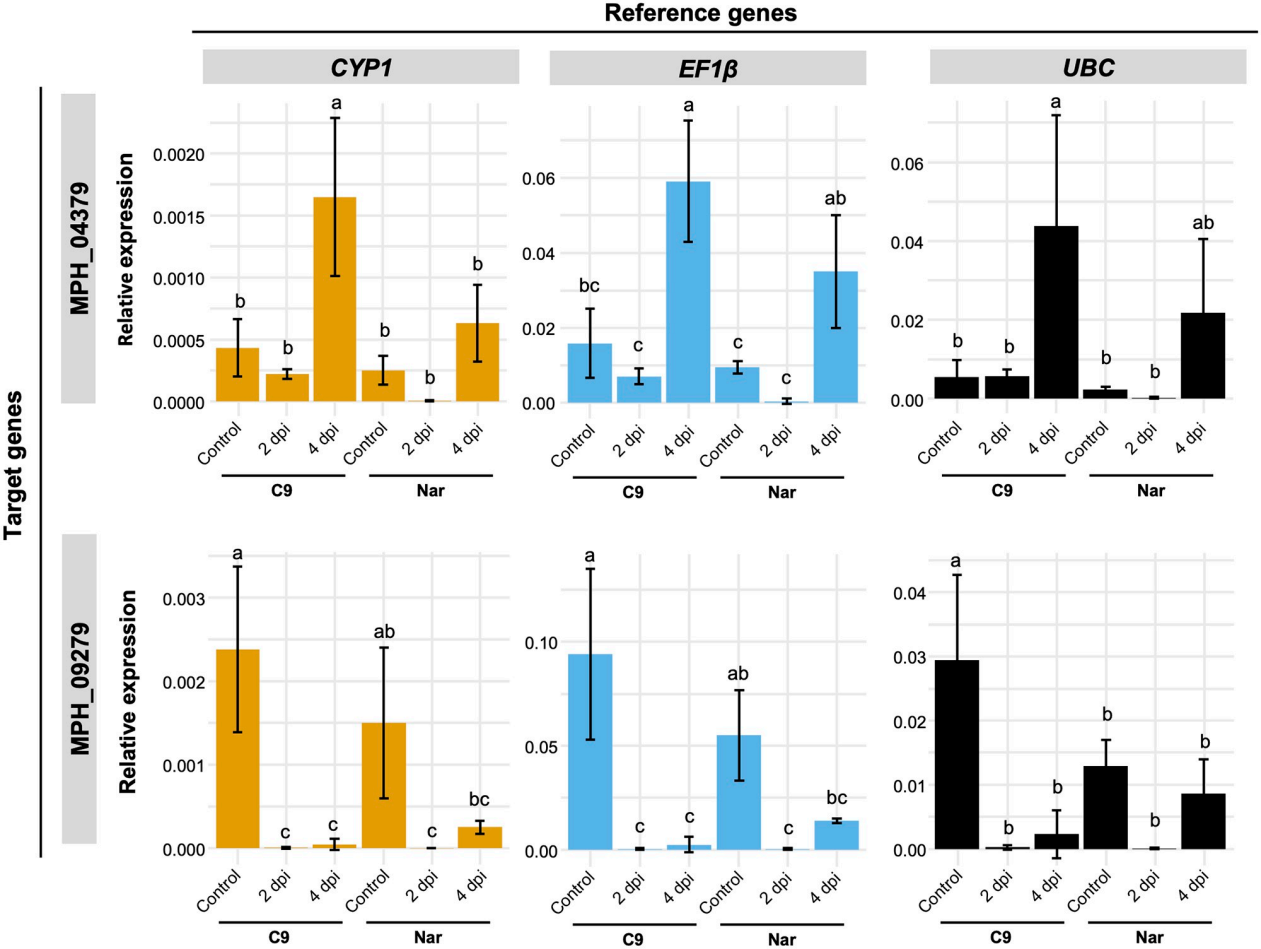
Expression of two cutinase genes of *M*. *phaseolina* during soybean root infection. Expression analysis of cutinase genes MPH_04379 (top) and MPH_09279 (bottom) was performed to validate the reference genes. RNA was extracted from soybean roots infected with isolate C9 and Nar at 2 and 4 dpi. *CYP1* (left) and *EF1β* (middle) were used as recommended internal controls, and *UBC* (right) was used as worst internal control. Data are mean ± standard deviation of at least three biological replicates. Different letters above the bars indicate significant differences at *P* < 0.05 (Tukey’s test).

In the case of *in vitro* root infection method, expression pattern was very similar when the target cutinase genes were normalized using *CYP1* and *EF1β* (Fig 3) indicating that both genes are suitable as reference gene. Although *UBC* was the least stable gene with this method, only a small impact was observed in the expression pattern of the target genes. These results indicate that MPH_04379 is induced at 4 days post-inoculation (dpi) and the expression level is higher in the isolate C9 than in Nar. In contrast, MPH_09279 was downregulated during the infection process.

**Fig 4:**
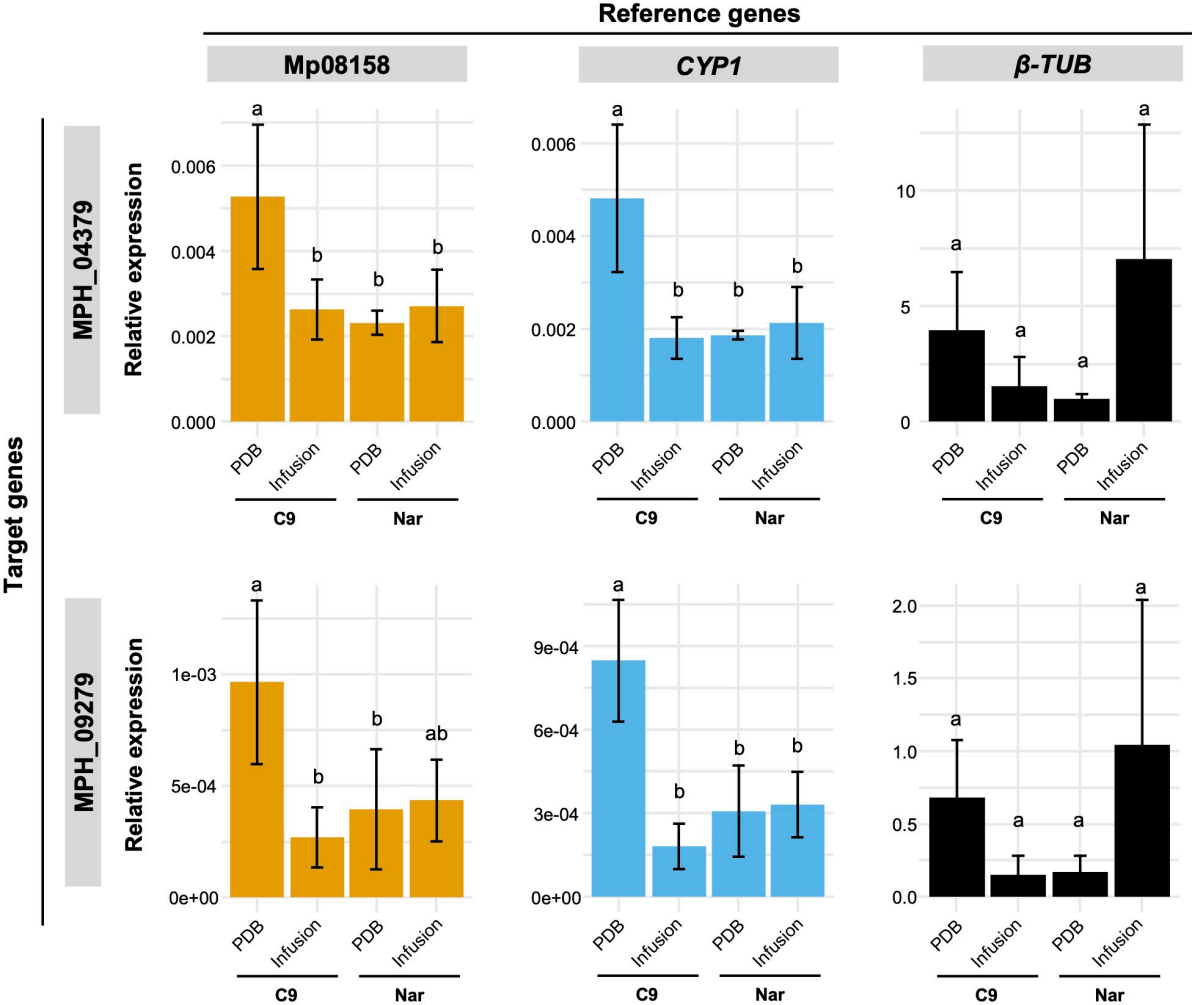
Expression of two cutinase genes of *M*. *phaseolina* grown in PDB with soybean leaf infusion. Expression analysis of cutinase genes MPH_04379 (top) and MPH_09279 (bottom) was performed to validate the reference genes. RNA was extracted from *M*. *phaseolina* isolates C9 and Nar grown in PDB medium and PDB with soybean leaf infusion. Mp08158 (left) and *CYP1* (middle) were used as recommended internal controls, and *β-TUB* (right) was used as worst internal control. Data are mean ± standard deviation of at least three biological replicates. Different letters above the bars indicate significant differences at *P* < 0.05 (Tukey’s test).

The results obtained from *M*. *phaseolina* grown in PBD with leaf infusion were similar to the *in vitro* root infection method. No difference was observed in expression pattern of the cutinase genes normalized with Mp08158 and *CYP1* (Fig 4). However, when the least stable gene, *β-TUB* was used for normalization, higher expression level of the target genes was observed in some of the replicates of the isolate Nar grown in PDB with leaf infusion, thereby resulting in higher standard deviation. Both cutinase genes presented similar expression pattern under this condition. No difference was observed between the expression level in the presence and absence of leaf infusion for the isolate Nar. On the other hand, the isolate C9 presented significantly higher expression in the absence of leaf infusion compared to that in the presence of leaf infusion.

**Fig 5:**
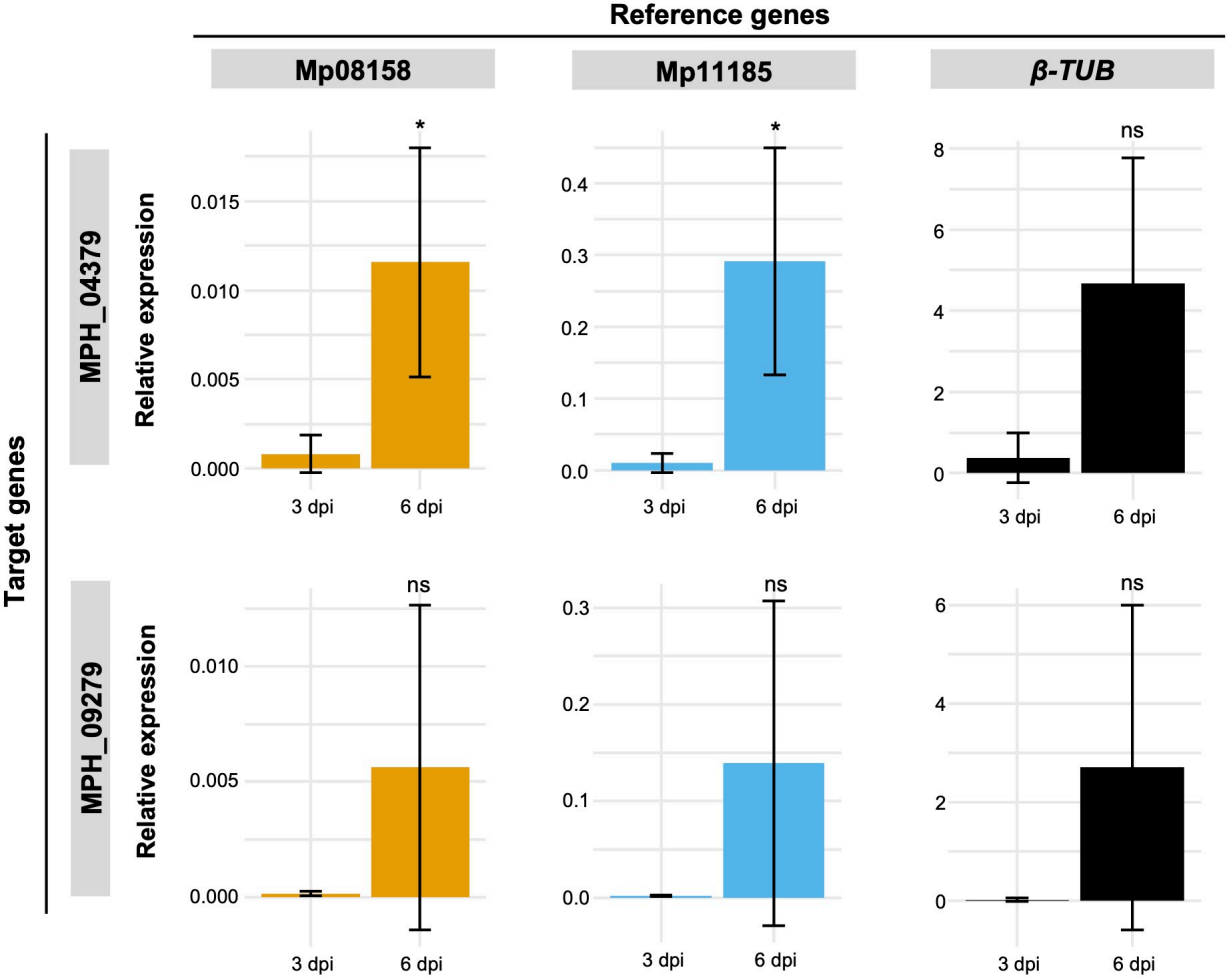
Expression of two cutinase genes of *M*. *phaseolina* during soybean stem infection. Expression analysis of cutinase genes MPH_04379 (top) and MPH_09279 (bottom) was performed to validate the reference genes. RNA was extracted from soybean stem infected with *M*. *phaseolina* isolate Nar at 3 and 6 dpi. Mp08158 (left) and Mp11185 (middle) were used as recommended internal controls, and *β-TUB* (right) was used as worst internal control. Data are mean ± standard deviation of at least three biological replicates. Student’s *t*-test was applied where *P < 0.05 and ns: not significant.

With cut-stem inoculation method, the expression pattern and variation of expression level among replicates were similar to the three genes (Mp08158, Mp11185 and *β-TUB*) used as normalizer (Fig 5). These results indicate that MPH_04379 was induced at 6 dpi and the expression of MPH_09279 tended to be higher at 6 dpi.

## Discussion

In the present study, suitable reference genes for the gene expression analysis of *M*. *phaseolina* were identified. This was done during the root and stem infection phases in soybean as well as in the culture media with and without adding soybean leaf infusion. For *in planta* expression analysis of the fungal genes, total RNA extract also contains plant RNA. Although cDNA is synthetized using the same amount of RNA (1 μg of total RNA per sample), the quantity of fungal RNA can differ among samples as fungal biomass increases with the progression of the infection. For samples collected at 2 dpi and 4 dpi in *in vitro* infected roots, no differences were observed in the Cq values (Fig 1), suggesting that the root infection initiated prior to 2 dpi. However, with cut-stem inoculation method, higher Cq values were observed in samples collected at 3 dpi than in those collected at 6 dpi, which can be associated with lower fungal colonization of stems at 3 dpi. This fungal biomass dependent Cq values (lower Cq values in heavily colonized tissues and vice versa) of candidate reference genes (such as Elongation factors, GAPDH, *β-*TUB, cytochrome b, UBC and polyubiquitin) were also observed in sunflower-*Puccinia helianthi* [35] and coffee-*Hemileia vastatrix* [36] pathosystems.

Our results show that there is no single (universal) gene suitable for normalization of target genes among different procedures. Thus, the reference gene of *M*. *phaseolina* should be determined for each experimental unit. Based on the results, *CYP1* can be recommended as reference gene for expression analysis using *in vitro* root infection method, which was consistent among the three algorithms. Besides *CYP1*, *EF1β* was also found to be quite stable. For *M*. *phaseolina* grown in PBD with leaf infusion, Mp08158 can be recommended as reference gene because of its consistent stability ranked by geNorm and NormFinder. With BestKeeper, Mp08158 was ranked among the three most stable genes. For cut-stem inoculation method, no single gene was consistently found as the best reference gene. Although Mp08158 and Mp11185 were the most stable genes in geNorm and NormFinder, they were found less stable based on the SD values obtained by BestKeeper.

Based on our results for three algorithms, *UBC* gene in the *in vitro* inoculation and *β-TUB* gene in cut-stem inoculation and growth in PDB with leaf infusion were found to be consistently least stable.

To test the selected reference genes, the expression of two cutinase genes were analyzed. In general, the use of least stable gene for normalization did not show great impact on the expression patterns of cutinase genes under our experimental conditions. A clear difference in expression pattern was observed in the fungus grown in PDB with and without leaf infusion. When most stable genes (Mp08158 and *CYP1*) were used, expression level of both cutinase genes was higher in C9 strain grown without leaf infusion than under other conditions. However, when least stable gene (*β-TUB*) was used, expression of cutinase genes was higher (although not significant) in Nar strain grown in PDB with leaf infusion. Different expression patterns caused by the use of most and least stable reference genes have been observed in other studies [37–40], indicating the importance of adequate selection of suitable genes for normalization under each experimental condition.

The importance of cutinase genes for plant-pathogen compatible interactions have been amply demonstrated. Disruption of some fungal cutinase genes can reduce virulence on host plants [41–45]. Nine cutinase genes have been identified in *M*. *phaseolina* genome [3], but their roles in pathogenesis were not studied. We analyzed the expression of two cutinase genes. The gene MPH_04379 of *M*. *phaseolina* was induced during soybean root and stem infection. As observed in some copies of cutinase genes of fungal species, it seems to be induced in the late-stage of infection [8, 44, 46]. On the other hand, MPH_09279 gene seems to be constitutively expressed as observed in some cutinase genes from *Curvularia lunata* [44] and *Magnaporthe grisea* [47]. In PDB medium, the presence of leaf infusion did not induce the two cutinase genes analyzed. During preparation of leaf infusion, plant tissue is removed by filtration. This suggest that the presence of plant tissue is important for induction of cutinase genes and that PDB medium (supplemented with leaf infusion) is not suitable for the analysis of these genes. Further studies are needed to evaluate which of the nine cutinase coding genes are essential for virulence of *M*. *phaseolina*.

The reference genes identified and validated in this study would be useful for gene expression analysis during host infection with *M*. *phaseolina*. This will enable identification of putative genes involved in pathogenesis and their further functional characterization.

## Supporting information

S1 Table Accession number of the RNA-seq reads used in this study. (PDF)

S2 Table Primers used in this study. (PDF)

S1 Fig *In vitro* root inoculation method. Root of soybean seedling was grown inside the Petri dish containing Hoagland’s solution solidified with agar, while the aerial part was grown outside the plate. The middle portion of primary root was inoculated with aqueous suspension of *M*. *phaseolina*.
(TIF)

S2 Fig Cut-stem inoculation method. A) At V2 stage, the main stem of soybean plants was cut 25 mm above the unifoliate node. A plug (of PDA medium colonized by *M*. *phaseolina*) containing pipette tip was placed on the cut-stem. White arrow indicate the plug inside the pipette tip. B) Three days after inoculation, the plug and pipette tip are removed from the cut-stem. Necrosis at the infected stem is observed.
(PDF)
