# Supplementary material for: Identification of reference genes and their validation for gene expression analysis in phytopathogenic fungus Macrophomina phaseolina
Source: PLoS One. 2022 Aug 5;17(8):e0272603. doi: 10.1371/journal.pone.0272603 (PMC9355225; doi:10.1371/journal.pone.0272603)
Supplement: S1 Table — (PDF) [file pone.0272603.s001.pdf]

S1 Table. Accession number of the RNA-seq reads used in this study.

BioProject	SRA	Condition	Reference
PRJNA326815	SRR5282579	*In vitro* Hoagland culture	Unpublished
	SRR5282606	*In vitro* Hoagland culture with plant extract	
PRJNA428521	SRR7548080	PDB with 4% NaCl at 28°C	(Burkhardt et al., 2019)
	SRR7548081	PDB with 2% NaCl at 28°C	
	SRR7548082	PDB pH 8 at 28°C	
	SRR7548083	PDB pH 3.5 at 28°C	
	SRR7548084	Strawberry crown-based media at 28°C	
	SRR7548085	PDB with 100 mM $(NH_4)_2SO_4$	
	SRR7548086	PDB at 35°C	
	SRR7548087	PDB under 24 h light at 28°C	
	SRR7548088	PDB at 15°C	
	SRR7548089	PDB at 28°C during 7 days	
PRJNA524935	SRR8648393	24 hpi of *Arabidopsis thaliana* Col-0 (replicate 1)	(Schroeder et al., 2019)
	SRR8648394	24 hpi of *A. thaliana* Col-0 (replicate 2)	
	SRR8648395	48 hpi of *A. thaliana* Col-0 (replicate 1)	
	SRR8648396	48 hpi of *A. thaliana* Col-0 (replicate 2)	
	SRR8648401	24 hpi of double mutant *ein2/jar1* (replicate 1)	
	SRR8648402	24 hpi of double mutant *ein2/jar1* (replicate 2)	
	SRR8648403	48 hpi of double mutant *ein2/jar1* (replicate 1)	
	SRR8648404	48 hpi of double mutant *ein2/jar1* (replicate 2)	

PDB: Potato dextrose broth.. Hpi: hours post-inoculation. Double mutant *ein2/jar1*: mutant of *A. thaliana* defective in ethylene and jasmonic acid signaling.

References

Burkhardt, A.K., Childs, K.L., Wang, J., Ramon, M.L., Martin, F.N., 2019. Assembly, annotation, and comparison of *Macrophomina phaseolina* isolates from strawberry and other hosts. BMC Genomics 20. https://doi.org/10.1186/s12864-019-6168-1

Schroeder, M.M., Lai, Y., Shirai, M., Alsalek, N., Tsuchiya, T., Roberts, P., Eulgem, T., 2019. A novel Arabidopsis pathosystem reveals cooperation of multiple hormonal response-pathways in host resistance against the global crop destroyer *Macrophomina phaseolina*. Sci. Rep. 9. https://doi.org/10.1038/s41598-019-56401-2
