# Supplementary material for: Identification of reference genes and their validation for gene expression analysis in phytopathogenic fungus Macrophomina phaseolina
Source: PLoS One. 2022 Aug 5;17(8):e0272603. doi: 10.1371/journal.pone.0272603 (PMC9355225; doi:10.1371/journal.pone.0272603)
Supplement: S2 Table — (PDF) [file pone.0272603.s002.pdf]

Table S2. Primers used in this study.

Genes	Locus tag	Sequence (5' to 3')	Efficiency	Product size
EF1β	MPH_03132	CAGACTCGCAGCTACATCGT<br>TCGAAAGAGGCGATGTGCTT	2.052	134 bp
UBC	MPH_13012	ATCAACTCCAACGGCAGCAT<br>TAAACGTGTGCGATCTCGGG	1.989	149 bp
EF1α	MPH_05497	CTGCCCCTCCAGGATGTCTA<br>TGCATCTCGACGGACTTGAC	1.904	143 bp
β-TUB	MPH_11587	AGCTCAACAACCCCAGCTAC<br>TTGCGCAGGTCAGAGTTCAG	1.947	109 bp
CYP1	MPH_07956	CAGAACGTTGCGAAGAAGCC<br>CTCGCCGTCCACAAACTCC	1.948	120 bp
CYP2	MPH_09910	CGATGTCGAGTCGTACAGGG<br>TGCTGGCCAGAATTGACGAT	1.877	105 bp
Mp05201	MPH_05201	CAGAAGCAGATTGACCGACA<br>GAACGCTCTTGTCGACCTTC	1.966	124 bp
Mp06465	MPH_06465	TGTTTGCGTTGAGAGATTCG<br>GAGGAGCTTGGTGATCTTGC	1.873	100 bp
Mp08158	MPH_08158	GGCGAGTCCAAGGTCTTCTA<br>GGCAGTCTTGTAAGCCTCGT	1.794	123 bp
Mp09417	MPH_09417	GCAGGTCGGCCAAAGACTAT<br>TAACCACACCCTCCAACGTG	1.889	130 bp
Mp09987	MPH_09987	TGCTTGACTCCCGCATCATT<br>CCTTAGCGAGGTCGGAATCC	1.889	120 bp
Mp11185	MPH_11185	AAGGGTGATCAGCTCATTGG<br>TTTTGCAGATCCTCCATTCC	2.088	107 bp
