# Supplementary figures and images for: Identification of reference genes and their validation for gene expression analysis in phytopathogenic fungus Macrophomina phaseolina
Source: PLoS One. 2022 Aug 5;17(8):e0272603. doi: 10.1371/journal.pone.0272603 (PMC9355225; doi:10.1371/journal.pone.0272603)

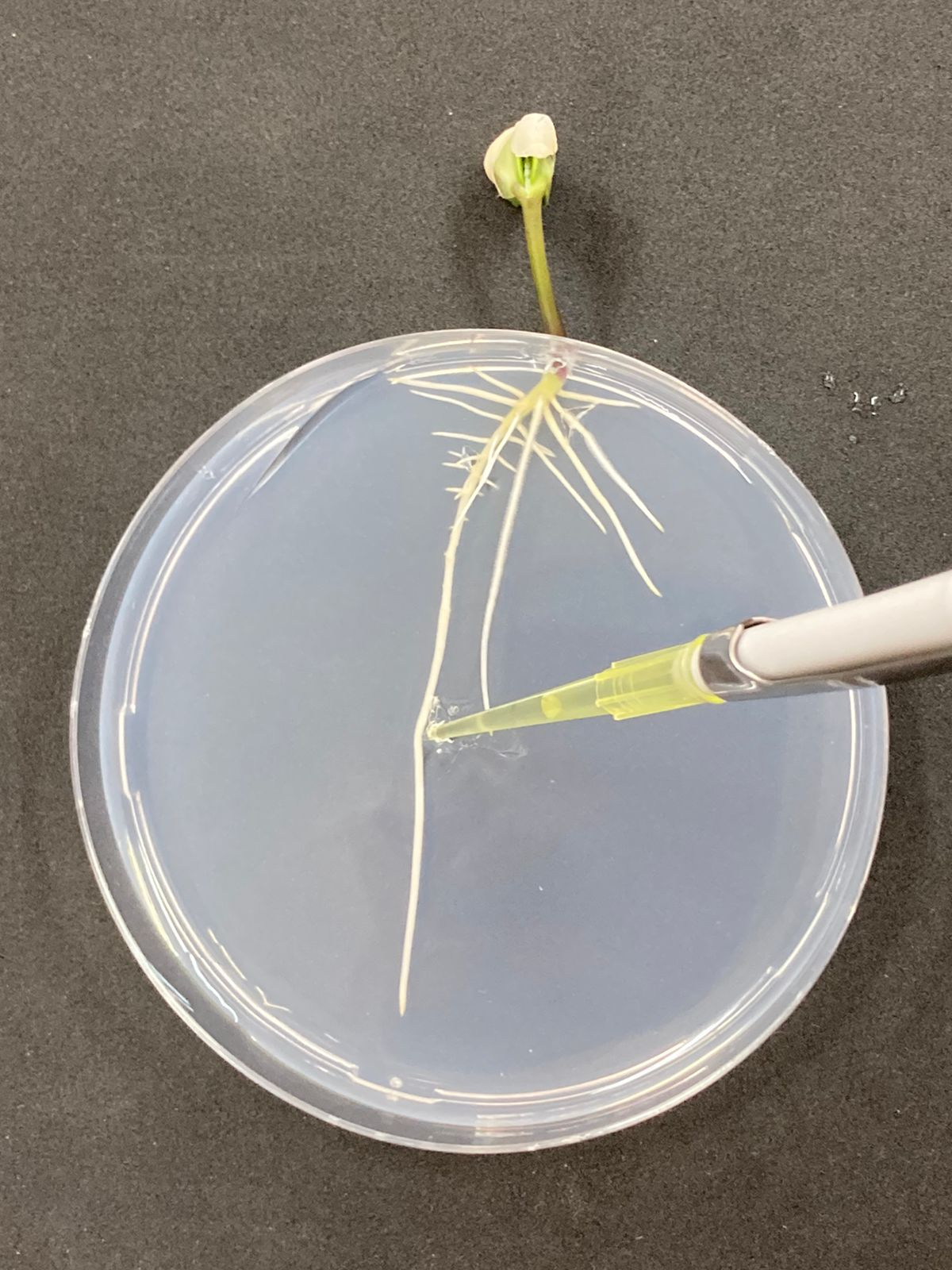

Supplement: S1 Fig — Root of soybean seedling was grown inside the Petri dish containing Hoagland’s solution solidified with agar, while the aerial part was grown outside the plate. The middle portion of primary root was inoculated with aqueous suspension of M. phaseolina. (TIF) [file pone.0272603.s003.tif]

A)

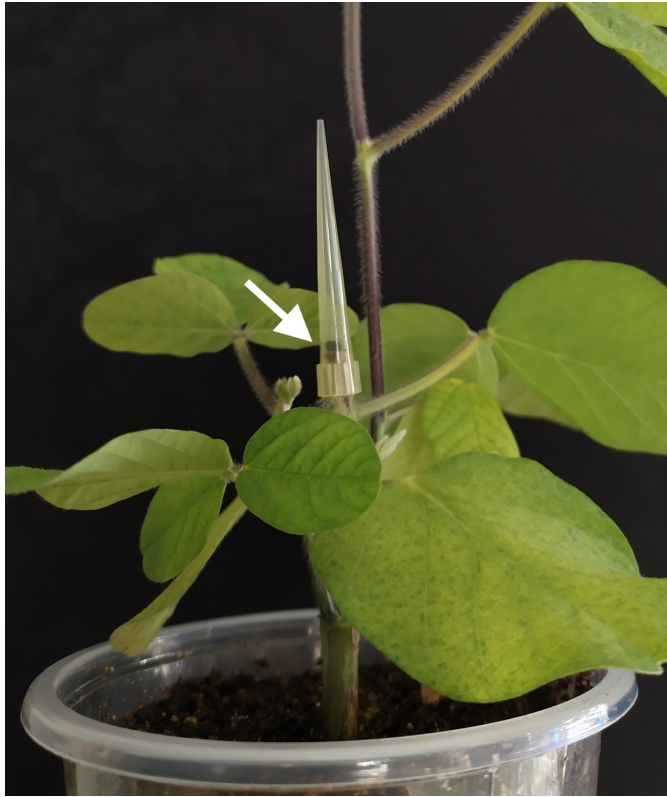

B)

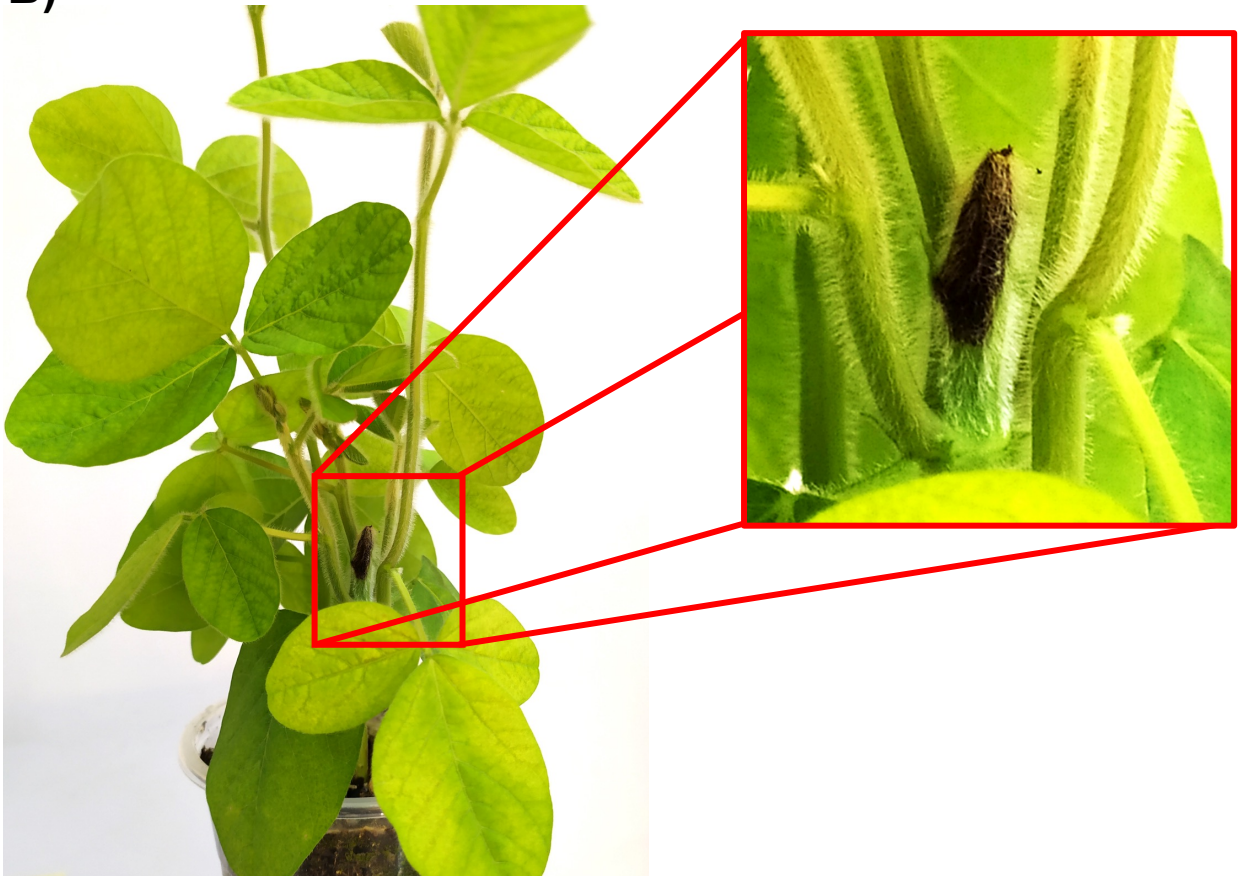

Supplement: S2 Fig — A) At V2 stage, the main stem of soybean plants was cut 25 mm above the unifoliate node. A plug (of PDA medium colonized by M. phaseolina) containing pipette tip was placed on the cut-stem. White arrow indicate the plug inside the pipette tip. B) Three days after inoculation, the plug and pipette tip are removed from the cut-stem. Necrosis at the infected stem is observed. (PDF) [file pone.0272603.s004.pdf]
